# Relationship between glycaemic control and lipid profile in type 2 diabetes mellitus patients in a low-resource setting

**DOI:** 10.11604/pamj.2022.41.281.33802

**Published:** 2022-04-07

**Authors:** Ifeyinwa Dorothy Nnakenyi, Emeka Francis Nnakenyi, Elijah Joshua Parker, Nene Orizu Uchendu, Emeka Godwin Anaduaka, Lawrence Uchenna Ezeanyika

**Affiliations:** 1 Department of Chemical Pathology, University of Nigeria, University of Nigeria Teaching Hospital, Ituku Ozalla, Enugu, Nigeria,; 2 Department of Morbid Anatomy, University of Nigeria, University of Nigeria Teaching Hospital, Ituku Ozalla, Enugu, Nigeria,; 3 Department of Biochemistry, University of Nigeria, Nsukka, Nigeria

**Keywords:** Cardiovascular disease, diabetes mellitus, dyslipidaemia, glycaemic control, lipid profile, low-resource setting

## Abstract

**Introduction:**

diabetes mellitus can lead to complications including cardiovascular disease (CVD). Glycated haemoglobin (HbA1C) is a test of glycaemic control in T2DM patients, and its association with CVD can be mediated through modulation of risk factors such as dyslipidaemia. It is suggested that correlation of HbA1c with blood lipids may enable its use as a dual marker for glycaemic status and dyslipidaemia. The aim of this study was to determine the relationship between glycaemic control and blood lipid concentrations in T2DM patients.

**Methods:**

a cross-sectional study of T2DM patients at Enugu, Nigeria. After obtaining informed consent, questionnaires were administered, and then venous blood was collected for determination of HbA1c and fasting lipid profile. Student T-test was used to compare mean results of two groups and Pearson correlation coefficient was used to determine relationships. A p-value <0.05 was considered to be statistically significant.

**Results:**

fifty -five (55) T2DM patients comprising of 24 females and 31 males, with mean±SD age 57±12 years were studied. Prevalence of patients with poor glycaemic control (HbA1c≥7%) was 34 (61.8%). More males (36.4%) than females (25.4%) had poor glycaemic control. There was a positive, statistically significant correlation between HbA1c and TC (r=0.406); Low-Density Lipoprotein Cholesterol (LDL-C) (r=0.409); and triglyceride (TG) (r=0.273), p<0.05. Correlation between HbA1c and HDL-C was negative (r=-0.269, p<0.05).

**Conclusion:**

the significant correlation between HbA1c and various lipid parameters may suggest the importance of glycaemic control as well as managing dyslipidaemia in the reduction of risk for CVD in T2DM patients, for which HbA1c may be used to monitor both, thereby reducing cost.

## Introduction

Diabetes mellitus is a group of metabolic diseases characterized by chronic hyperglycaemia resulting from defects in insulin secretion, insulin action, or both. Diabetes mellitus causes about 5% of all deaths globally each year [1], as the chronic hyperglycaemia is associated with long-term damage, dysfunction, and failure of various organs, especially the eyes, kidneys, nerves, heart, and blood vessels due to cardiovascular disease (CVD). Cardiovascular morbidity and mortality is high in the majority of patients with diabetes, in particular with Type 2 diabetes mellitus (T2DM), who are at a 2- to 4- fold higher risk of cardiovascular mortality compared with non-diabetic subjects [2]. Up to 90% of CVD may be preventable if established risk factors are avoided [3]. Patients with T2DM often exhibit an atherogenic lipid profile, characterized by high plasma levels of triglyceride (TG), total cholesterol (TC), low-density lipoprotein cholesterol (LDL-C), but low level of high-density lipoprotein cholesterol (HDL-C) [4]; as well as increased free fatty acids, increased small dense LDL (sdLDL), which greatly increases their risk for CVD via the process of atherosclerosis. Although hyperglycaemia was associated with atherosclerotic lesion initiation, addition of increasing amounts of cholesterol led to dyslipidaemia, which was the major factor in atherosclerosis progression, independent of hyperglycaemia [5].

Worsening of glycaemic control deteriorates lipid and lipoprotein abnormalities as a growing body of evidence suggests that dyslipidaemia is secondary to insulin resistance or factors closely related to insulin resistance, such as adiposity [6]. Increased free fatty acid flux secondary to insulin resistance and increased proinflammatory adipokines and cytokines from enlarged adipose tissue may be the underlying determinants of this interrelationship [7]. The combination of hyperglycaemia and dyslipidaemia produces an enhanced atherogenic environment within the circulation which accelerate the progression to atherosclerosis [8].

Glycated hemoglobin (HbA1c) is a routinely used marker for long-term glycaemic control [9]. It is a form of haemoglobin that is chemically linked to a sugar by the process called glycation and reflects the weighted mean plasma glucose concentration during the preceding 2-3 months. It is relatively insensitive to short-term lifestyle changes. It is used as a tool for monitoring glycaemic control and quality of care in patients with diabetes [10]. There is an established log-linear correlation between HbA1c and microvascular complications in diabetic individuals [11], but the relationship between HbA1c and macrovascular disease is unclear. Apart from classical risk factors like dyslipidaemia, elevated HbA1c has now been regarded as an independent risk factor for CVD in subjects with or without diabetes [12], although there is still some controversy over its use as a prognostic marker for CVD outcomes and/or mortality. It is estimated that there is an 18% increased risk of CVD for each 1% rise in absolute HbA1c levels in the diabetic population [13]. A study from Japan showed a significant association between HbA1c and CVD [14], and it has been suggested this association can be mediated through modulation of CVD risk factors such as dyslipidaemia [15]. Therefore, some studies have reported that HbA1c could potentially be utilized as a possible biomarker for predicting dyslipidaemia as well as CVD [16,17].

Thus, it would be beneficial to correlate the level of HbA1c in diabetic patients with their lipid profiles and risk ratios, with an aim to use HbA1c as a dual marker for glycaemic status and dyslipidaemia in T2DM patients, thereby reducing the cost of investigating for both conditions especially in low-resource settings. Therefore, the aim of this study was to determine the relationship between glycaemic control and lipid profile in T2DM patients in our low-resource setting.

## Methods

**Study design and location:** this was a descriptive, cross-sectional study of the relationship between glycaemic control and lipid profile in T2DM patients conducted at the diabetic outpatient clinic at University of Nigeria Teaching Hospital (UNTH), which is a tertiary health institution that serves semi-urban and rural communities in Enugu State, Nigeria. The study was conducted from July to December 2019.

**Ethical considerations:** ethical approval was obtained from the Health Research and Ethics Committee of UNTH, Enugu (*NHREC/05/01/2008B-FWA00002458-1RB00002323*) to ensure the research was performed according to the Declaration of Helsinki. Informed consent was obtained from the subject after explaining the details of the study in English and/or native Igbo language and they confirmed their understanding of the research and their participation. Confidentiality was ensured.

### Study population

**Inclusion criteria:** adult patients (18 years and above) who had been diagnosed with T2DM, who may or may not have commenced anti-diabetic medication were included in the study.

**Exclusion criteria:** paediatric patients, pregnant women, hypertensive and type 1 DM patients were excluded.

**Sample size determination:** the study sample size was determined based on the formula:


$$n=\frac{z^{2}pq}{d^{2}}$$

Where n = sample size, z= critical value at 95% confidence level usually set at 1.96, p=prevalence (3.7% prevalence rate of DM in southeast Nigeria) [18], q= 1-p, d= imprecision of 5%. Inputting the variables:


$$n={\left(1.96\right)}^{2}\times 0.037\times 0.963/{\left(0.05\right)}^{2}=54.7\approx 55$$

Therefore, a total sample size of 55 was studied.

**Sampling technique:** participants were selected from the outpatient register by simple random sampling using a table of random numbers.

**Preparation of questionnaires:** the questionnaire was newly developed by the researchers. It began with an informed consent page, that provided potential participants with information on the study and its aims, why the patient was selected to participate and requirements from him/her, potential benefits and risks to him/her, what will happen to the information obtained and the name and address of the researcher. Patients having understood these terms and agreed to participate in the study were administered the questionnaire. The content of the questionnaire included mixed open and closed ended questions to obtain information on the blood glucose and lipid monitoring practice of the patients. The questionnaire layout was as follows: demographic (age and sex), socioeconomic (educational level and occupational status), diabetes history (diagnosis and treatment), blood glucose monitoring history, blood lipid profile monitoring history, and past medical history of cardiovascular events.

**Testing and validation of questionnaires:** prior to the commencement of the study, the questionnaires are administered to ten T2DM patients who were not included in the study. This was to ensure standardization and proper comprehension of the questions asked in a bid to prevent bias. But psychometric testing was not performed.

**Patient preparation:** in order to eliminate the confounding factor from recent diet and effect modifiers to the lipid profile results, the selected patients were instructed to return the next day after an overnight fast i.e. no ingestion of food or drink 10 - 12 hours after last night meal, for the study.

**Data collection:** questionnaires were administered to participants to obtain information on basic demographics, relevant medical and drug history. Thereafter, specimen collection ensued.

**Specimen collection:** antiseptic preparation of the antecubital vein was effected with methylated spirit swabs. A tourniquet was applied to gain access to the vein, and 8 mls of venous blood was collected by venipuncture using a 10 ml syringe. Five mls of blood was dispensed into a plain tube for lipid profile assay and 3 mls was dispensed into an ethylene diamine tetra-acetic acid (EDTA) tube for the HbA1c assay.

**Specimen processing:** the venous blood specimens were taken to the laboratory for processing. Those in the plain tube were allowed to clot and retract for 1 hour, and then centrifuged at 4000 rpm for 5mins at room temperature to separate the supernatant (serum) which was analyzed for lipid profile concentrations which consisted of: TC, HDL-C, LDL-C and TG. The EDTA samples were mixed homogenously for 5mins, then analyzed for HbA1c concentration. Haemolysed, icteric and lipaemic samples were not analyzed.

**Assay methods and calculations:** enzymatic methods were used for the determination of TC [19], TG [20] and HDL-C [21]. Low-density lipoprotein cholesterol concentration was determined using the Friedewald equation [22], which is as follows: LDL-C = TC - HDL-C - (TG/2.22) to be expressed in mmol/l. HbA1c levels were determined using the boronate affinity chromatography method [23] and reported in percentage (%). Estimated average glucose concentration was calculated using the formula: (1.59 x HbA1c%) - 2.59 [24]. Atherogenic Index was determined from the lipid profile results using the equation: log10 of serum (TG/HDL-C) [25], while Grover´s risk ratios were also determined from the lipid profile results using the equations: LDL-C/HDL-C; TC/HDL-C [26].

**Determination of undesirable lipid levels:** the National Cholesterol Education Program Adult Treatment Panel III defined the following undesirable lipid levels [4]: hypercholesterolaemia is defined as TC greater than 200 mg/dl (5.2 mmol/L), high LDL-C when the value is over 100 mg/dl (3.3 mmol/L), hypertriglyceridemia when TG is greater than 150 mg/dl (1.7 mmol/L) and low HDL-C has a value less than 40 mg/dl (1.0 mmol/L). Dyslipidaemia was defined as the presence of one or more abnormal serum lipid concentrations above.

**Statistical analysis:** data were entered into Microsoft Excel Spreadsheet (IBM, USA) and analyzed using Statistical Package for the Social Sciences (SPSS) software version 20 (Chicago IL, USA). Data were presented as mean ± standard deviation. Student T-test was used to compare the mean results between two groups. Prevalence was determined by number of affected patients over total number of patients and presented as frequency (percentage %). Pearson´s correlation coefficient (r) was used to determine the correlation between HbA1c and each of the lipid profile parameters, as well as the cardiovascular risk ratios. Linear regression was used to determine association between HbA1c and each of the lipid profile parameters, as well as the cardiovascular risk ratios. A p-value of <0.05 was considered to be statistically significant.

## Results

Sixty-eight T2DM patients were recruited for the study, but 55 T2DM patients participated and completed the study. The others (13) did not show up for the follow-up visit after the overnight fast to commence the study. The participants comprised of 24 (43.6%) females and 31 (56.4%) males, who had mean ± standard deviation ages of 59 ± 12 years and 55 ± 12 years respectively. The highest frequency of patients was between ages 51 - 60 years. There was no statistical difference between any of the biochemical results of the females versus the male patients (p >0.05). The number of patients with poor long-term (over the previous three months) glycaemic control, defined as HbA1c greater than 7% [27] were 34 (61.8 %). More males (36.4%) than females (25.4%) had poor glycaemic control.

The biochemical parameters of the patients were categorized by HbA1c values and compared (Table 1), which revealed that there was no statistical difference between both groups except for the HbA1c and estimated average glucose results. Table 2 shows the prevalence of patients with features of dyslipidaemia, which was defined using cut-off values for undesirable lipid concentrations [4]. More males than females had features of dyslipidaemia, except LDL-C which was equal in both. More patients with poor glycaemic control (HbA1c ≥ 7.0%) had features of dyslipidaemia (Table 3). The atherogenic index of the patients revealed equal number of female and male patients had values in the 'safe' negative range -14 (25.5%), but more males had values in the 'unsafe' positive range 17 (30.9%).

**Table 1:** comparison of biochemical parameters categorized by patient's HbA1c

Biochemical parameter (unit)	HbA1c ≥ 7.0% (n = 34)	HbA1c < 7.0% (n=21)	p-value
HbA1c (%)	9.00 ± 1.78	5.28 ± 0.57	< 0.05*
Total cholesterol (mmol/L)	5.64 ± 1.11	5.28 ± 1.07	> 0.05
Triacylglycerol (mmol/L)	1.22 ± 0.49	1.07 ± 0.38	> 0.05
HDL-cholesterol (mmol/L)	1.10 ± 0.32	1.15 ± 0.38	> 0.05
LDL-cholesterol (mmol/L)	3.98 ± 1.16	3.64 ± 1.20	> 0.05
Estimated average glucose (mmol/L)	11.72 ± 2.83	6.61 ± 0.91	< 0.05*
Atherogenic index	0.03 ± 0.26	-0.04 ± 0.21	> 0.05
TC/HDL	5.71 ± 2.41	5.10 ± 2.01	> 0.05
LDL/HDL	4.12 ± 2.13	3.63 ± 1.85	> 0.05

**Table 2:** prevalence of patients with features of dyslipidaemia

Undesirable lipid concentrations	Females	Males	Total
Total cholesterol ≥ 5.2 mmol/L	16 (29%)	17 (30.9%)	33 (59.9%)
Triacylglycerol ≥ 1.7 mmol/L	1 (1.8%)	7 (12.7%)	8 (14.5%)
HDL - cholesterol < 1.0 mmol/L	7 (12.7%)	16 (29%)	23 (41.7%)
LDL - cholesterol ≥ 3.3 mmol/L	18 (32.7%)	18 (32.7%)	36 (65.4%)

**Table 3:** distribution of patients according to their glycaemic and dyslipidaemic status

Undesirable lipid concentrations	HbA1c ≥ 7%	HbA1c < 7%
Total cholesterol ≥ 5.2 mmol/L	24 (43.6%)	9 (16.4%)
Triacylglycerol ≥ 1.7 mmol/L	6 (10.9%)	2 (3.6%)
HDL - cholesterol < 1.0 mmol/L	14 (25.5%)	9 (16.4%)
LDL - cholesterol ≥ 3.3 mmol/L	24 (43.6%)	12 (21.8%)

There was a positive correlation between HbA1c and TC which was statistically significant (r = 0.406, p < 0.05). Likewise, the correlation between HbA1c and LDL-C was positive and statistically significant (r = 0.409, p < 0.05). As well as the positive, statistically significant relationship between HbA1c and triacylglycerol (r = 0.273, p < 0.05). The relationship between HbA1c and HDL-C was inverse, giving a negative correlation, which was statistically significant (r = - 0.269, p < 0.05). The weak to moderate correlations observed (r = 0.2 to 0.4) may be due to the limited sample size of this study, as a larger sample size should produce stronger correlation coefficients. Linear regression analysis was significant for each of the lipid parameter. The relationship between HbA1c and atherogenic index was positive and statistically significant. Similarly, the correlation between HbA1c and TC/HDL-C, as well as LDL-C/HDL-C were positive and statistically significant (p < 0.05).

## Discussion

The association of Diabetes mellitus and CVD has been well described over the past few decades. There is high risk of CVD in people with T2DM, with CVD deaths being the top killer in this population [28], for which hyperglycaemia and dyslipidaemia play significant roles in the pathogenesis of CVD. This study observed a mean ± SD HbA1c level of 7.8 ± 2.12%, and about 62% of the patients had levels ≥ 7.0% suggesting poor glycaemic control over the previous three months. This is because the longer hyperglycaemia persists; there is the opportunity for glycation of proteins such as haemoglobin. This prevalence was consistent with findings of Bhattacharjee *et al*. who found poor glycaemic control in 60% of their patients [29].

There was a high proportion of dyslipidaemia in this study evidenced by hypercholesterolaemia-TC (60%), LDL-C (65.4%), hypertriglyceridaemia (14.5%), and low HDL-C (41.7%). This is consistent findings by Yan *et al*. [30]. It is suggested that insulin resistance has a central role in the development of diabetic dyslipidemia. One of the causes is increased free fatty acid release from insulin-resistant fat cells. If the glycogen stores are adequate, these free fatty acids promote TG production which further stimulates apolipoprotein B (apo-B) and Very Low Density Lipoprotein (VLDL) [31]. The apo-B regulates the enzymatic activity of lipoprotein lipase and cholesterol ester transport protein which transfers cholesterol from HDL to TG-rich lipoproteins with reciprocal transfer TG to HDL resulting in a particle that is rapidly catabolized and cleared from plasma resulting in low levels [32]. This study´s prevalence of dyslipidaemia differed from findings by Davis *et al*. which were much lower [33]. They reported their study subjects had levels of TC > 200 mg/dl (25.2%), LDL-C > 130 mg/dl (17.6%), TG < 150 mg/dl (26.9%), and HDL-C ≤ 35 mg/dl (9.3 %) [33].

In this study, the correlation of HbA1c and lipid profile in T2DM was evaluated, and it was discovered that there was a positive relationship between HbA1c and TC, TG and LDL-C, as well as with the atherogenic index, TC/HDL-C and LDL-C/HDL-C, which were all statistically significant (p < 0.05). Conversely, there was a negative relationship with HDL-C, and it was also statistically significant (p < 0.05). This was similarly reported by Hussain *et al*. who observed that the there was a significant positive correlation between HbA1c, TC, TG, LDL-C and LDL-C/HDL-C ratio [34]. Their correlation between HbA1c and HDL-C was negative and was statistically non-significant. A study in India reported similar findings of direct significant correlation between HbA1c and TC, TG, LDL-C, TC/HDL-C, LDL-C/HDL-C and there was inverse correlation between HbA1c and HDL [29], while another study reported that HbA1c levels had a significant direct relationship with TC, TG and LDL-C but not with HDL-C [35].

The implication of these relationships is that addressing glycaemic control may result in better lipid levels. Wägner *et al*. showed that the improvement in glycaemic control from HbA1c of 10.54 ± 2.05% to HbA1c of 7.01 ± 0.63% (p <0.0005) after a follow-up period of 3.5 months resulted in a significant reduction in LDL-C - from 3.62 ± 1.15 to 3.34 ± 1.02 mmol/L (p <0.05), and apo B from 1.17 ± 0.29 to 1.07 ± 0.25 g/L (p <0.01), with increase in LDL particle size from 25.10 ± 0.31 to 25.61 ±0.53 nm (p <0.005) in T2DM patients who had LDL phenotype B at baseline [36]. Thus, HbA1c can provide valuable information besides its primary role in monitoring long-term glycemic control.

Linear regression analysis indicated HbA1c was a predictor of TC, LDL-C, TG, HDL-C. This was consistent with findings by Hussain *et al*. who reported that HbA1c was a predictor of hypercholesterolemia, LDL-C and TG via linear regression analysis [34]. Similarly, Alzahrani *et al*. reported that their linear regression results indicated that HbA1c values were associated with TG (p= 0.020) and were independent of age, BMI, TC, LDL-C, HDL-C and fasting plasma glucose levels [37]. These suggest that apart from a reliable glycaemic index, HbA1c may also be used as a predictor of dyslipidaemia, and in turn early diagnosis of dyslipidaemia can be used as a preventive measure for the development of CVD in patients with T2DM.

The biochemical parameters in this study were observed to be higher in the T2DM patients with poor glycaemic control (HbA1c ≥ 7.0%), than those with good glycaemic control (HbA1c < 7.0%), but the difference was not statistically significant except for HbA1c and estimated average glucose (p < 0.05). This is likely because worsening glycaemic control deteriorates lipid and lipoprotein abnormalities of diabetes mellitus [38]. These results were found to be consistent with findings by Alzahrani *et al*. who reported that in both groups, no significant differences were found in any of the parameters other than TGs (p = 0.020) and HbA1c (p <0.001) [37]. Conversely, the results were not consistent with findings of Hussain *et al*. who found that patients with HbA1c value greater than 7.0% had significantly higher value of cholesterol, LDL-C, and LDL-C/HDL-C ratio compared with patients with an HbA1c value up to 7.0% [34]. Also, Bhattacharjee *et al*. reported that patients with HbA1c ≥ 7% had a significant increase in TC, LDL-C, TG, TC/HDL-C and LDL-C/HDL-C ratio and a decrease in their HDL-C levels as compared to patients with HbA1c < 7.0% [29]. It is important to target better glycaemic control, because improving glycaemic control may substantially reduce the risk of cardiovascular events. It has been projected that a decrease in the HbA1c value by 0.2% could lower mortality by 10% [34].

The patients with poor glycaemic control (HbA1c ≥ 7.0%) had unsafe, positive atherogenic index, while those with good glycaemic control (HbA1c < 7.0%) had safer, negative atherogenic index. The atherogenic index values range from negative to positive with zero closely corresponding to the LDL diameter of 25.5nm. Positive values are associated with higher risk for coronary heart disease, while negative values signify low risk for coronary heart disease [25]. Similarly, the risk ratios TC/HDL-C and LDL-C/HDL-C, which have been described as the best lipid-related predictor of future cardiovascular event [26], were higher in the poor glycaemic control patients than those with good glycaemic control. This may imply that the former group (HbA1c ≥ 7.0%) has a higher risk for cardiovascular morbidity and mortality [39]. Other risk ratios such as TG/HDL ratios have been related to T2DM and also predicts for cardiovascular disease [40].

This study did not observe any statistically significant difference between the biochemical parameters of the females versus the males (p > 0.05). This finding was not consistent with findings by Alzahrani *et al*. who reported that their T2DM females had significantly higher values for HbA1c (p = 0.009), triglycerides (p <0.001), HDL-C (p = 0.002) and LDL-C (p < 0.001) compared to the males [37]. Although, their study had a much higher ratio of females to male (2.2: 1) than this one.

**Limitations:** although the sample size of this study was deemed adequate on calculation, it is quite small, and this may limit the power of the study, therefore our work should be considered as a pilot study. Further studies based on larger patient numbers are still needed on the role of HbA1c as a marker for dyslipidaemia in T2DM. These studies may also predict the occurrence of cardiovascular disease among T2DM using HbA1c. In the development of the questionnaire used in this study, psychometric testing was not done which may have concerns for reliability, validity and fairness; but the questionnaire was tested and validated on similar study participants prior to use to address those concerns.

## Conclusion

The results of this study showed a significant correlation between HbA1c and various lipid parameters risk ratios, and may suggest the importance of glycaemic control as well as managing dyslipidaemia, in a bid to further reduce the risk for cardiovascular disease in patients with T2DM. It is being proposed that HbA1c can be used as an important marker to predict dyslipidaemia in patients with T2DM in addition to glycaemic control, and the results of this study support this. The implication is that HbA1c will be a cost-effective test, as it will lessen the cost of investigating for both conditions, especially in low-resource settings.

### What is known about this topic

- *Hba1c is a measure of glycaemic control and chronic hyperglycaemia;*
- *Diabetes mellitus may lead to an atherogenic lipid profile;*
- *Hyperglycaemia and dyslipidaemia are risk factors for cardiovascular disease*.

### What this study adds

- *Hba1c correlates well with lipid parameters and risk ratios;*
- *Hba1c may be used to monitor for both glycaemic control and dyslipidaemia*.
